# Common viral infections seed regionally distinct resident memory T cells in the human CNS

**DOI:** 10.21203/rs.3.rs-10885314/v1

**Published:** 2026-10-18

**Authors:** Pamela Rosato, Hanna Degefu, Tyler Searles, Tiffany Chen, Shawn Musial, Jordan Isaacs, Sierra Kleist, Shizhao Yang, Myles Ford, Fred Kolling, Alexander Skorput, Chun-Chieh Lin, George Zanazzi, Jennifer Hong, Li Song

## Abstract

T cells persist in the central nervous system (CNS) and can drive both protection and neurological disease. How these cells are organized in humans and what they recognize is largely unknown. Here, we profiled CD8⁺ T cells across anatomically distinct CNS regions, obtained through on-site autopsies and temporal lobe resection surgeries, using single-cell RNA sequencing, paired T cell receptor sequencing, and DNA-barcoded tetramers. Resident memory T cells (T _RM_ ) specific for Epstein-Barr virus, cytomegalovirus, influenza A, and SARS-CoV-2 were identified across CNS compartments. Anatomical location was the strongest correlate of T _RM_ cell state, with leptomeningeal cells adopting a cytokine-poised T _RM_ program, whereas brain T _RM_ cells were transcriptionally restrained. Cells of the same clonotype spanned tissues yet adopted local transcriptional states. Viral specificity added another layer of T _RM_ heterogeneity with *GZMK/GZMA-* expressing EBV-specific populations and interferon-stimulated gene signatures in SARS-CoV-2 and Influenza A-specific cells. The human CNS thus harbors regionally distinct CD8 ^+^ T _RM_ shaped by common viral exposures.

